# When rare diseases do not appear as a single entity

**DOI:** 10.1515/almed-2025-0193

**Published:** 2026-07-16

**Authors:** Isabel Solares, Estibaliz Alegre, Inmaculada Colina, Juan Jose Gavira, Beatriz Alvarez de Sierra, Sara Llorente-Gonzalez, Álvaro Gonzalez

**Affiliations:** Rare Disease Unit, Internal Medicine Department, Clinica Universidad de Navarra, Madrid, Spain; Biochemistry Department, Clínica Universidad de Navarra, Pamplona, Spain; Navarra Institute for Health Research (IDISNA), Pamplona, Spain; Department of Cardiology, Clínica Universidad de Navarra, Pamplona, Spain; Department of Radiology, Cancer Center, Clinica Universidad de Navarra, Madrid, Spain; Retinal Pathologies and New Therapies Group, Experimental Ophthalmology Laboratory, Department of Ophthalmology, Clinica Universidad de Navarra, Pamplona, Spain

**Keywords:** propionic acidemia, mitochondrial disease, Leber hereditary optic neuropathy

## Abstract

**Objectives:**

The coexistence of multisystemic involvement in a young adult raises the need to consider rare metabolic and mitochondrial disorders. We report a case of late-onset propionic acidemia (PPA) that may have contributed to unmasking Leber hereditary optic neuropathy (LHON), an extremely infrequent association.

**Case presentation:**

A 43-year-old woman presented with acute biventricular heart failure following a viral infection. Evaluation revealed severe dilated cardiomyopathy, newly diagnosed type 2 diabetes mellitus, and bilateral sensorineural hearing loss. Genetic testing for cardiomyopathy was unremarkable. Two months later, she developed sudden bilateral blindness due to optic neuropathy. Extended genetic analysis identified a pathogenic *RDH12* variant consistent with LHON and two *POLG* variants. Progressive renal dysfunction and long QTc interval prompted metabolic investigation. Urine organic acids showed marked accumulation of propionate-related metabolites, and plasma acylcarnitines revealed elevated C3 and low free carnitine, confirming late-onset PPA. The pattern of cardiac dysfunction, renal impairment, and hearing loss was consistent with chronic multisystem toxicity of PPA.

**Conclusions:**

In this patient, mitochondrial dysfunction from PPA may have precipitated the clinical expression of LHON, a condition with incomplete penetrance. This exceptional coexistence highlights that rare diseases may overlap and that unexplained multisystem findings warrant comprehensive metabolic and genetic evaluation.

## Case presentation

A 43-year-old woman presented with 5 days of evolution of biventricular heart failure triggered by a viral respiratory infection in the previous week. As a previous medical history, the patient had an episode of metabolic acidosis of unexplained cause at the age of 2 years. She is the eldest of two sisters of non-consanguineous parents.

A transthoracic echocardiogram was performed, which revealed a previously unknown dilated cardiomyopathy with severe ventricular dysfunction (Left Ventricular Ejection Fraction: 23 %). During the admission, type 2 diabetes mellitus (T2DM) and moderate bilateral sensorineural hearing loss were also diagnosed. A filtered genetic panel within a whole exome sequencing (WES) for dilated cardiomyopathy was requested in which no clinically relevant genetic variants were identified. The patient presented an adequate clinical evolution under depletive treatment. Nevertheless, two months later she presented an episode of sudden blindness in the context of bilateral optic neuropathy.

## Discussion

Due to the association of T2DM in a young patient, sensorineural hearing loss, dilated cardiomyopathy with severe dysfunction of unknown etiology and sudden amaurosis in relation to bilateral optic neuropathy, it was decided to optimize the genetic study for mitochondrial disease, which identified:

– Variant c.210dupC (p.Arg71GlnfsTer12) in heterozygosity in the *RDH12* gene, classified as pathogenic. Pathogenic mutations in this gene have been associated with Leber Hereditary Optic Neuropathy (LHON) [1].
– Variants c.752C>T (p.Thr251Ile) and c.1760C>T (p.Pro587Leu), in heterozygosity, classified as pathogenic in the *POLG* gene.

**Figure 1:**
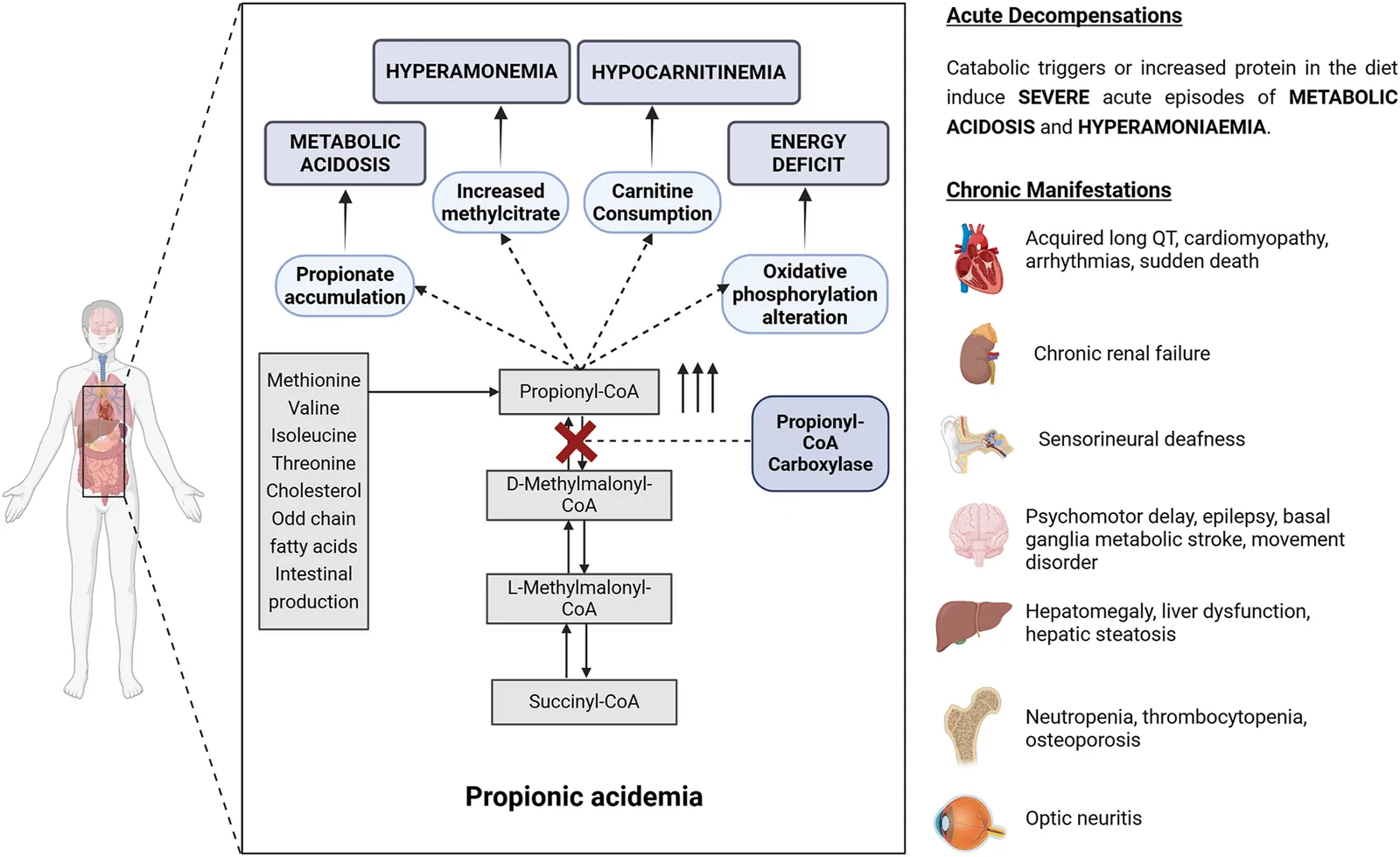
Representation of the pathophysiology and clinical manifestations of propionic acidemia.

The *RDH12* mutation justified the sudden visual loss in the context of LHON. However, although the phenotypic spectrum of mitochondrial depletion symptoms associated with *POLG* mutations is highly variable, muscle involvement, epilepsy, ptosis/ophthalmoplegia, and leukoencephalopathy are characteristic [2]. Therefore, a study aimed to characterize *POLG* clinical spectrum was carried out with blood tests, brain and muscle Magnetic Resonance Imaging (MRI), and electromyography. All these tests were normal (Table 1, Figure 1). Furthermore, family segregation was carried out, confirming that *POLG* mutations were CIS-variants inherited from her mother.

**Table 1:** Biochemical results of the patient.

Analyte	Units	Result		Reference interval
		May 2023	June 2023	
Glucose	mg/dL	**112**	**102**	74–100
Hemoglobin A_1c_	%	**6.8**	**7.1**	≤6.49
	mmol/mol	**51**	**54**	≤48
Insulin	µU/mL	**4.5**	**60.2**	2.6–24.9
HOMA-R		1.23	15.16	≤2.5
Bilirubin, total	mg/dL		0.42	≤1.2
AST	UI/L		19	≤35
ALT	UI/L	**38**	17	≤35
ALP	UI/L	**62**	47	35–104
GGT	UI/L		17	≤40
LDH	UI/L		183	135–225
Sodium	mmol/L	140	142	136–145
Potassium	mmol/L	4.2	3.9	3.5–5.0
Chloride	mmol/L	103	107	98–107
Calcium	mg/dL		**10.1**	8.6–10.0
Creatinine	mg/dL	0.9	**1.3**	0.5–0.9
eGFR CKD-EPI	mL/min/1.73 m^2^	82	**52**	≥60
Vitamin B12	pg/mL	219	268	197–771
Folate	ng/mL		11.5	3.89–26.8
Albumin, urine	mg/dL	**34.4**	0.8	≤1.5
	mg/g creatinine	**137.3**	3.4	≤30

Bold: abnormal results.

At the 2-month follow-up visit, a decline in renal function was observed, characterized by worsening glomerular filtration in the absence of proteinuria. The electrocardiogram showed a long QTc (474 ms) and cardiac MRI confirmed biventricular dilated cardiomyopathy, negative for ischemia in the stress study.

Although possible, this pattern of multisystem organ damage is not typical of mitochondrial disease, in which renal involvement most commonly manifests as albuminuria and/or low–molecular-weight proteinuria, and cardiac involvement most frequently presents as hypertrophic cardiomyopathy [3], 4]. Therefore, an alternative possibility is that the patient has a disorder characterized by the accumulation of toxic substances, leading to multiorgan damage (ear, kidney, heart, etc.) and secondary mitochondrial dysfunction, which may have unmasked LHON – an entity known for its low clinical penetrance.

### Case resolution

Urine organic acids assayed by gas chromatography/mass spectrometry, showed the significant accumulation of propionate, propionylglycine, methylcitrate and tiglilglycine. The acylcarnitine profile in plasma shows a significant increase in propionylcarnitine (C3) and a decrease in free carnitine (C0). Based on these metabolic alterations, the patient was diagnosed with propionic acidemia (PPA) with late onset. The WES was reviewed, filtering for the *PCCA* and *PCCB* genes, and only two variants of uncertain significance in the *PCCA* gene (c.674G>A and c.637+5G>A) were identified.

PPA is an inborn error of metabolism of genetic origin due to mutations in *PCCA* or *PCCB*, which produces a deficiency of the mitochondrial enzyme propionyl-CoA carboxylase (EC 6.4.1.3). PPA has an estimated incidence of 1:150,000 but debut in adulthood is extremely rare [5]. Organic acid analysis in urine reveals a characteristic pattern with increased 3-hydroxypropionate, methylcitrate, propionylglycine, and propionylcarnitine that persists between seizures.

The symptoms of PPA can be divided into acute crises and chronic manifestations. Acute episodes are characterized by metabolic acidosis due to the accumulation of propionate that is triggered by catabolic situations such as infections or surgery, or by an increase in protein intake. The inhibition of urea cell cycle regulatory enzyme N-acetylglutamate synthase by the accumulation of propionyl-CoA may also cause secondary hyperammonemia [6]. The accumulation of propionate also entails the consumption of carnitine and the generation of other products derived from the alternative oxidation of propionate that have the capacity to inhibit different enzymatic systems, such as mitochondrial oxidative phosphorylation. The deficiency in energy production and the toxic effect of accumulated substrates can lead to the development of different chronic organic complications [7]. PPA is frequently associated with cardiac disease, such as dilated cardiomyopathy and acquired long QT syndrome. Arrhythmias may lead to severe events, including sudden cardiac arrest [8]. The exact mechanism of kidney damage is unclear but maleic acid could be implicated due its cellular toxic effects and interference on mitochondrial energy production [9]. Hearing impairment seems to be secondary to the accumulating metabolites on the KvLQT1/KCNE1 channel protein [10]. Patients with PPA can exhibit a wide range of neurological manifestations, liver complications and immunology disorders [9]. Optic neuropathy is associated with late-onset propionic acidemia, with a reported prevalence between 11 % and 25 % of patients, and it may present most commonly in an insidious and progressive manner, or acutely during metabolic crises. However, the patient never had a metabolic decompensation in adulthood. Findings include optic nerve atrophy, scotomas, and abnormalities in visual evoked potentials, and it has been documented in both pediatric cohorts and young adults with late-onset forms of the disease [11], [12], [13], [14], [15]. The identification of the *RDH12* variant has been reported in the literature as being responsible for Leber hereditary optic neuropathy, with clinical manifestations of sudden, irreversible amaurosis, a presentation that more closely resembles that observed in our patient [1], 16].

Finally, at present, this patient would have been diagnosed at birth in Spain, as propionic acidemia is included in the national newborn screening program. In contrast, at the time of her birth 43 years ago, neonatal screening had not yet been implemented. This circumstance highlights the critical role of newborn screening in enabling early diagnosis of inborn errors of metabolism.

## Conclusions

In the specific case of our patient, besides PPA, she also carried a mutation associated with LHON disease. These mutations have a known incomplete penetrance, so not all mutation carriers will develop vision loss. The mechanisms involved have not been completely elucidated, but complex genetic and environmental factors seem to play a role in the clinical penetrance of the disease [9]. The mitochondrial dysfunction secondary to her PPA may have contributed to the clinical expression of LHON, presenting with acute visual loss. Considering the combined prevalence of PPA and LHON, the probability of their coexistence is less than one in 100 million. Although these diseases are very infrequent, the presence of one rare disease does not exclude the existence of another, and both clinicians and laboratory professional should work together to unmask them.

## Lessons learned

– Multi-organ disease without an unexplained cause in a young patient should raise suspicion of a possible underlying genetic metabolic disease.
– Given the specificity of the biochemical tests necessary for the diagnosis of these diseases, diagnostic suspicion must be high.
– Organic acidemias, such as PPA, produce an increase in organic acids in biological fluids, acute metabolic consequences and chronic manifestations, such us cardiomyopathy, deafness or chronic renal failure.
– PPA produces an alteration in oxidative phosphorylation that can favor manifestations secondary to mitochondrial dysfunction.
– The presence of one rare disease does not exclude the coexistence of another; multidisciplinary collaboration between clinicians and laboratory specialists is crucial to reach an accurate diagnosis.
